# Estimation of Pollutant Emissions in Real Driving Conditions Based on Data from OBD and Machine Learning

**DOI:** 10.3390/s21196344

**Published:** 2021-09-23

**Authors:** Néstor Diego Rivera-Campoverde, José Luis Muñoz-Sanz, Blanca del Valle Arenas-Ramirez

**Affiliations:** 1 Machine-Engineering Division, Escuela Técnica Superior de Ingenieros Industriales—ETSII, Universidad Politécnica de Madrid, 2 José Gutierrez Abascal Street, 28006 Madrid, Spain; joseluis.munozs@upm.es; 2 Grupo de Investigación en Ingeniería del Transporte, Universidad Politécnica Salesiana, Calle Vieja 1230 and Elia Liut, 010105 Cuenca, Ecuador; 3 Instituto Universitario de Investigación del Automóvil Francisco Aparicio Izquierdo—INSIA-UPM, Escuela Técnica Superior de Ingenieros Industriales—ETSII, Universidad Politécnica de Madrid, 2 José Gutierrez Abascal Street, 28006 Madrid, Spain; blanca.arenas@upm.es

**Keywords:** emission model, OBD emissions model, real driving emissions, machine learning, internal combustion engine

## Abstract

This article proposes a methodology for the estimation of emissions in real driving conditions, based on board diagnostics data and machine learning, since it has been detected that there are no models for estimating pollutants without large measurement campaigns. For this purpose, driving data are obtained by means of a data logger and emissions through a portable emissions measurement system in a real driving emissions test. The data obtained are used to train artificial neural networks that estimate emissions, having previously estimated the relative importance of variables through random forest techniques. Then, by the application of the K-means algorithm, labels are obtained to implement a classification tree and thereby determine the selected gear by the driver. These models were loaded with a data set generated covering 1218.19 km of driving. The results generated were compared to the ones obtained by applying the international vehicle emissions model and with the results of the real driving emissions test, showing evidence of similar results. The main contribution of this article is that the generated model is stronger in different traffic conditions and presents good results at the speed interval with small differences at low average driving speeds because more than half of the vehicle’s trip occurs in urban areas, in completely random driving conditions. These results can be useful for the estimation of emission factors with potential application in vehicular homologation processes and the estimation of vehicular emission inventories.

## 1. Introduction

Internal combustion engines (ICE) of automobiles are a major source of pollution in urban areas, contributing significantly to the deterioration of air quality in cities [1], which causes a serious problem given that, according to the United Nations Organization in 2016, 54.5% of the world’s population lives in urban areas [2]. Private vehicle trips are the main cause of fuel wastage and unnecessary CO_2_ emissions. These show inefficiency in three domains: driver behavior, route selection and traffic management [3], wherein parameters such as deficient deceleration, incorrect selected gear and engine speed, excessive speed and acceleration, congestion, poorly synchronized traffic signals, inefficient transfer route choice and lack of knowledge and motivation are highlighted [4,5].

CO_2_ emissions are certified under standardized conditions, which are very different from real driving conditions, so when there are not enough data available, simulation can be used [6]. Several stochastic models determine the influence of various variables on fuelconsumption during road driving, such as speed and the presence of road features and characteristics that determine stop/start sequences, like stop signals, speed reducers, driving curves and topography. These situations affect eco-driving [7], whilst in urban driving [8], it has been determined that fuel consumption is strongly influenced by vehicular congestion.

Pollutant emissions, consumption and performances generated by ICE are closely related to the gradient of the road and the driving style [9,10], especially the gear change strategy of the driver [11,12]. Beckx and Vlieger [13] studied the influence of gear change on emissions and fuel consumption by using simulations, confirming that consumption and the emissions of CO_2_, CO and HC decrease when ascendant gear changes are made, while NOx emissions show slight variations; emphasizing that this is a theoretical study based on non-realistic speed profiles, they therefore propose, as [14] does, the use of real driving cycles to obtain results that are close to reality. Brundell and Erickson [15] determined that speed, acceleration and gear choice have an influence on pollutant emissions and consumption, so for this purpose they used two different mechanistic models of instantaneous emissions in four vehicles [16], determining that, in order to estimate representative emission factors, it is necessary to refine and validate the models used in transitory stages of operation specially. Boulter et al. and Zöldy et al. [17,18] determined that pollutant emissions depend on vehicle-specific factors, such as model, weight, fuel type, technology level and travelled distance, and operational factors, such as speed, acceleration, gear selection, road gradient and environmental temperature, so therefore all emission models must take these factors into consideration. The estimation of pollutant emissions in the laboratory over dynamometer chassis and in adjusted driving cycles is lower than the one determined in real driving cycles, as concluded by [4,10], respectively, and are lower than the ones obtained in the RDE. Kurtyka and Pielecha [19] conclude in the same way, emphasizing that the difference in results between the dynamometer tests and in the RDE are due to traffic conditions and driving style.

A novel methodology to estimate pollutant emissions is presented in this article, using as input data the driving variables of the vehicle, such as: throttle position, manifold absolute pressure, vehicle and engine speed coming from the OBD (onboard diagnostic) and the gear used by the driver, obtained through the application of clustering techniques such as K-means and classification trees. Additionally, information from the global positioning system (GPS) was used to determine the altitude above sea level. With the aim of creating a pollutant emissions estimation model, a real driving emissions (RDE) test was carried out in a route where both emissions and OBD data were obtained. With these data, an ANN (artificial neural network) was trained, which had been validated with the data obtained in a second RDE test, confirming the validity of the emission estimator. Finally, this estimator was applied to a data set of 1218.9 km of real driving, whose results were compared to those obtained in the IVE model and RDE test, showing evidence of similar results. The methodology applied in this paper has the advantage of evaluating vehicle performance without the need of using PEMS for long driving tests. The closeness of the results compared reveals the power of the models adjusted to the data obtained in RDE, which are those established in the regulation [20]. This model can be used for the estimation of emission factors with potential applications in vehicular homologation processes and the estimation of vehicular emission inventories by means of real driving tests of short duration, avoiding therefore long measurement campaigns and the prolonged use of PEMS, as shown in [17].

## 2. Materials and Methods

### 2.1. Methodology for the Estimation of Emission Gases under Real Driving Conditions

According to [21], the emissions of a vehicle must be evaluated under normal driving conditions, which excludes laboratory tests using standardized driving cycles, while computerized models for estimating emissions require databases with characteristics of: vehicle fleet, fuels, environmental conditions and geographic location [8]. Currently, the Cuenca Mobility Company (EMOV-EP) estimates the emissions inventory based on the MOBILE6-Mexico model [22], which considers only vehicles manufactured in the USA without including those manufactured in the European Union and Asia [23] and which also considers types of fuels, environmental and traffic conditions different from those of the city of Cuenca in its database. Therefore, the proposed methodology is novel, since as far as the authors know, it would be the first contribution in Ecuador for the estimation of polluting emissions of one of the most common vehicles in real driving and environmental conditions.

**Figure 1:**
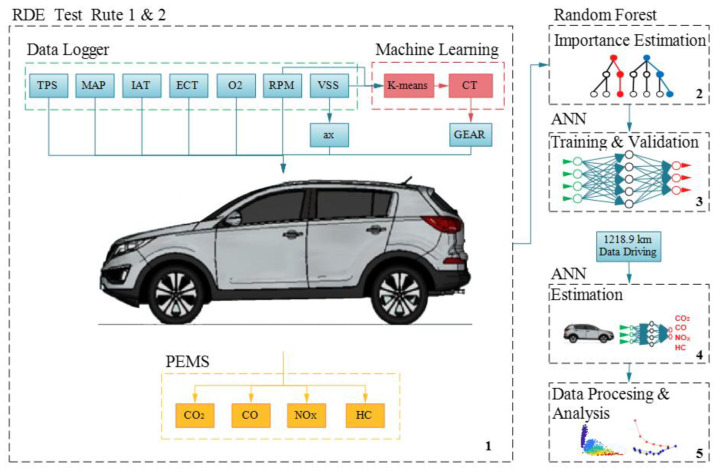
Methodology and procedure proposed for the estimation of gas emissions from the OBD data.

To estimate exhaust gasses emissions using real driving parameters, the following steps that make up the new methodology are proposed and are the same ones that are represented in Figure 1:

1. Real driving and emission data collection;
  - Estimation of the selected gear during driving;
  - Estimation of the relative importance of each measured variable;
2. Training and validation of the neural network with the most significant variables of Route 1 and validation of the ANN calculated by applying on Route 2;
3. Application of the data set of 1218.9 km to the validated ANN;
4. Processing and presentation of results.

The procedure for each of the steps proposed in this methodology is detailed below.

### 2.2. Driving and Emission Real Data Collection

#### 2.2.1. Test Vehicle

The vehicle used in the route tests is a Kia Sportage 2018 model, which is the best-selling SUV in Ecuador according to the Automobile Company Association of Ecuador, 2018 [24]. The vehicle has a DOHC 2.0 L engine, 6-speed manual transmission and 18,720 km of travelled distance according to the tachometer and with all the maintenance operations recommended by the manufacturer at the beginning of the measurement campaign.

#### 2.2.2. Portable Emission Measurement System PEMS

A gas analyser Brain Bee AGS-688, which works by means of the NDIR method (nondispersive infrared), was used to measure CO_2_ [%], CO [%] and HC [ppm], and using an electrochemical cell to measure O_2_ [%] and NO_X_ [ppm], emissions were measured on a dry basis. The equipment collected data at a frequency of 10 Hz and was powered by a battery that was independent of the test vehicle, as established in Euro 6 RDE [20]. The equipment had a calibration certificate by ISO/IEC 17025 using span gas according to ISO 6145, valid at the time of sampling.

#### 2.2.3. Data Logger

Operational parameters of the vehicle were obtained through OBD together with the GPS information using Freematics ONE+ data logger at a frequency of 15.15 Hz and stored on a micro-SD card. Fuel consumption was measured using AIC Fuel Flow Master 5004. The operating and driving parameters are shown in Table 1.

**Figure 2:**
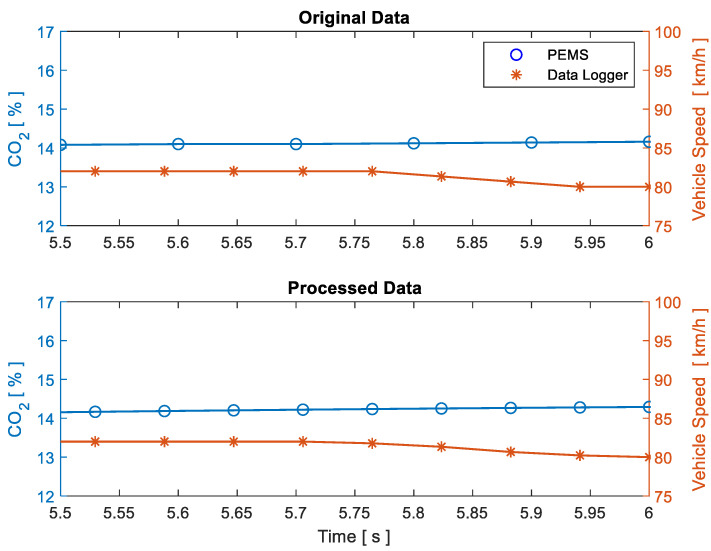
Signal processing from PEMS and data logger.

The data logger recorded the information in CSV format, generating a separate file for each driving cycle. This file was vectorised to obtain a time series matrix. The Savitzky-Golay algorithm was subsequently applied to each variable in order to eliminate outliers and soften the discretisation of the measured data [25]. PEMS and data logger recording equipment showed different sampling frequencies, therefore, a re-sampling and re-measuring algorithm was created to obtain compatible vectors regarding size and synchronization. ANNs were used for this purpose, which increased the number of PEMS samples, making them compatible with the number of data logger records, as shown in Figure 2.

#### 2.2.4. Test Routes

**Figure 3:**
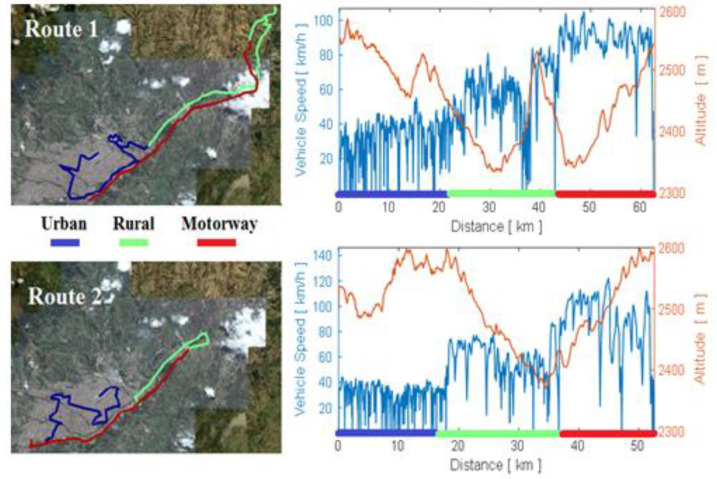
Route, altitude and speed during the RDE test.

In order to analyse the performance of the test vehicle during the application of the real driving emission (RDE) test [20], two different routes were proposed: Route 1 and Route 2. The data set obtained in Route 1 was divided into 70% of the data for training, 15% for validation and the remaining 15% for ANN testing. The data set obtained in Route 2 was used for a double cross-validation of the adjusted ANN. The route is chosen for the data collection for the RDE test in the city of Cuenca–Ecuador–South America, which has its urban area in the city centre and the rural area in the Panamerica Norte road, and the main motorway is the Cuenca–Azogues motorway, as shown in Figure 3.

The environmental temperature during the test was 14 °C with no rain or strong winds; the weight of the vehicle including two passengers and a full fuel tank was 1719.5 kg. The vehicle was driven with windows closed, without activating the air conditioning and under minimal traffic conditions. Fuel (92 octane) was used according to the recommendations of the manufacturer. The RDE trip characteristics are shown in Table 2.

#### 2.2.5. Estimation of the Selected Gear

**Figure 4:**
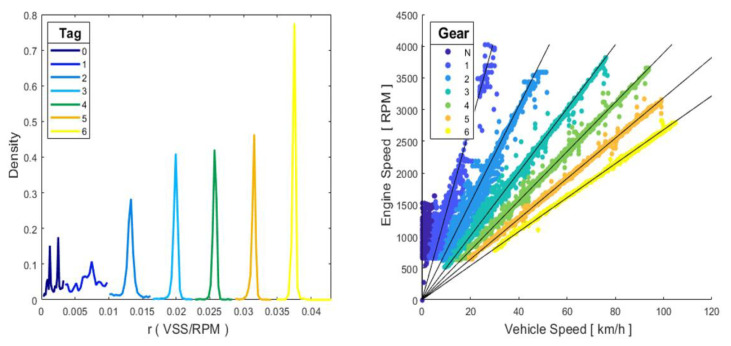
Labels obtained through K-means algorithm. CT-generated results.

The test vehicle, like most manual transmission vehicles, does not have a selected gear sensor; therefore, it must be determined from the OBD-obtained data. A state-of art-review evidences the lack of an automatic method to infer the gear used in every moment of the trip [11,26] and can estimate the selected gear from the engine speed and wheel speed achieved from the CAN Bus data, identifying the RPM/u ratio within the previously determined intervals. Values that did not fall within the above-mentioned intervals were considered gear changes. Therefore, this paper presents a methodology that allows, based on the vehicle speed and the engine speed and by applying machine learning, the determination of the gear with a high degree of certainty (over 99.5% accuracy). The K-means algorithm was applied to the data obtained in the RDE test, to the r_i_ = VSS_i_/RPM vector specifically, which generated a label for each of the 7 groups obtained from their centroids [27], and the groups generated corresponded to each one of the 6 vehicle gears and to the neutral position. A classification tree (CT) was trained with the label obtained, which was applicable to all sampled driving cycles, given that the use of gears in a driving cycle is random, making it necessary to draw upon supervised learning [28]. The generated tree had 7 splits and had a 99.5% effectiveness rate, from which the matrix G_i_ = [G_0_, G_1_, G_2_, G_3_, G_4_, G_5_, G_6_] was obtained and whose elements take value 1 depending on the gear selected in sample i. The labels obtained and CT results are detailed in Figure 4.

#### 2.2.6. Pollutant Estimation

**Figure 5:**
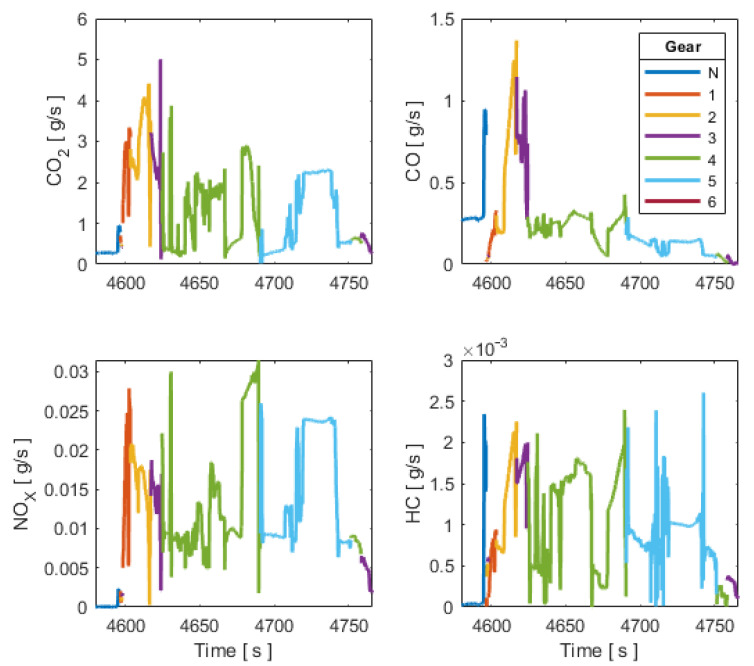
Tailpipe CO_2_, CO, HC and NOX emissions.

From the volumetric concentrations of pollutants in the exhaust gases, the mass flow rates of each pollutant are determined by using the procedure described in [20]. The exhaust mass flow rate ${\dot{m}}_{ex}\;\left[\;g/s\;\right]\;$is estimated from Equation (1).

(1) $${\dot{m}}_{ex}={\dot{m}}_{in}+{\dot{m}}_{f}$$

where ${\dot{m}}_{in}$ is the air mass flow estimated from the parameters obtained from OBD, and ${\dot{m}}_{f}$ is the fuel flow measured by the rotary piston flowmeter located in the fuel-line. Emissions are measured on a dry basis and must therefore be corrected by Equations (2) and (3).

(2) $$C_{wet,\;j}=k_{w,j}\;C_{dry,j}$$

(3) $$k_{w}=\frac{1.008}{1+0.005\alpha ({∁}_{CO_{2}}+{∁}_{CO})}$$

where $C_{wet,\;j}$ is the concentration on a wet basis of the pollutant *j* in volume; $\;C_{dry,j}$ is the concentration of the pollutant on a dry basis; $k_{w}$ is the correction factor from dry to wet bases; α is the molar ratio of hydrogen, and $\;{∁}_{CO_{2}}+{∁}_{CO}$ are the concentrations on a dry basis of CO_2_ and CO, respectively. The instantaneous mass emissions of each pollutant ${\dot{m}}_{j,\;i}\;\left[g/s\right]$ are obtained by Equation (4).

(4) $${\dot{m}}_{j,\;i}=c_{j,\;i}\mu_{j,\;i}\;{\dot{m}}_{ex,i}\;10^{-3}\;$$

where *i* is the measuring number; $c_{j,}$ is the instantaneous concentration of the gas component in [ ppm ], and $\mu_{j\;}\;$is the ratio between the density of each component and the overall exhaust density. In [20] they are determined to be $\mu_{CO2}=0.001518,\;\;\mu_{CO2}=0.000966,\;\mu_{NOx}=0.001587,\;\;\mu_{HC}=0.000499$. The instantaneous emission values of the vehicle can be obtained based on this estimation, as shown in Figure 5.

**Figure 6:**
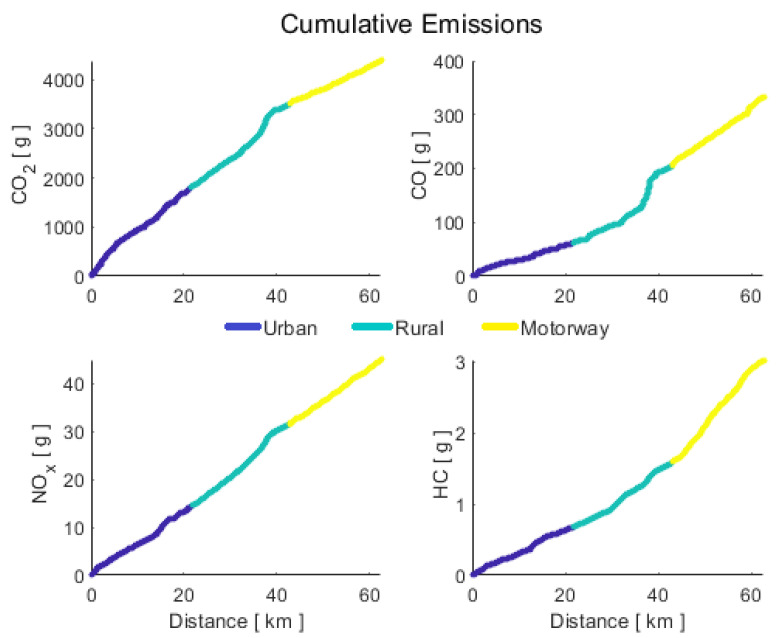
Cumulative emissions during RDE test.

The emission of each pollutant $m_{j}$ [g] in the driving cycle is equal to the summation of its instantaneous emissions regarding time, as shown in Equation (5).

(5) $$m_{j}=\overset{n}{\underset{i=1}{\sum }}{\dot{m}}_{j,i}\;∆t$$

where $\dot{m}$ is the instantaneous mass flow of the pollutant *j*; *n* is the number of samples in the data set, and ∆t is the sampling time, which is equal to 0.1 s. The cumulative emissions are detailed in Figure 6.

The emission factors $F_{j,k}\;$per each pollutant [g/km] in section *k* of the RDE were determined by Equation (6).

(6) $$F_{j,k}=\frac{m_{j,k}}{s_{k}}$$

where $m_{j,k}$ is the mass of the pollutant *j,* and s is the travelled distance in section *k* of the RDE. *k* assumes the values of *u, r* and *m* for urban, rural and motorway sections, respectively. The results obtained are shown in Table 3.

The mass flow of each pollutant ${\dot{m}}_{j,\;i,G}$, the total mass per trip $m_{j,G}$ and the total travelled distance $s_{j,G}\;$ per each gear selected G is estimated by:

(7) $${\dot{m}}_{j,\;i,G}=c_{j,\;i}\mu_{j,\;i}\;{\dot{m}}_{ex,i}\;G_{i}\;10^{-3}$$

(8) $$m_{j,G}=\overset{n}{\underset{i=1}{\sum }}{\dot{m}}_{j,i}G_{i}\;∆t$$

(9) $$s_{j,G}=\overset{n}{\underset{i=1}{\sum }}3.6\;VSS_{j,i}G_{i}\;∆t$$

(10) $$F_{j,G}=\frac{m_{j,G}}{s_{j,G}}$$

### 2.3. Estimation of the Relative Importance of Each Measured Variable

To optimise the training process of the ANNs, the use of the most representative or influential variables was prioritised based on the importance of predictor variables provided by the random forest (RF) technique that matched in the selection according to the Gini criterion. RF is based on multiple classification and regression trees (CART) to reduce dimensionality problems in the prediction of variables, therefore improving the accuracy and stability of the model obtained from the average of the results of the individual CART models applied to data sets wherein not all the variables involved are considered because they are randomly chosen in each CART [26].

**Figure 7:**
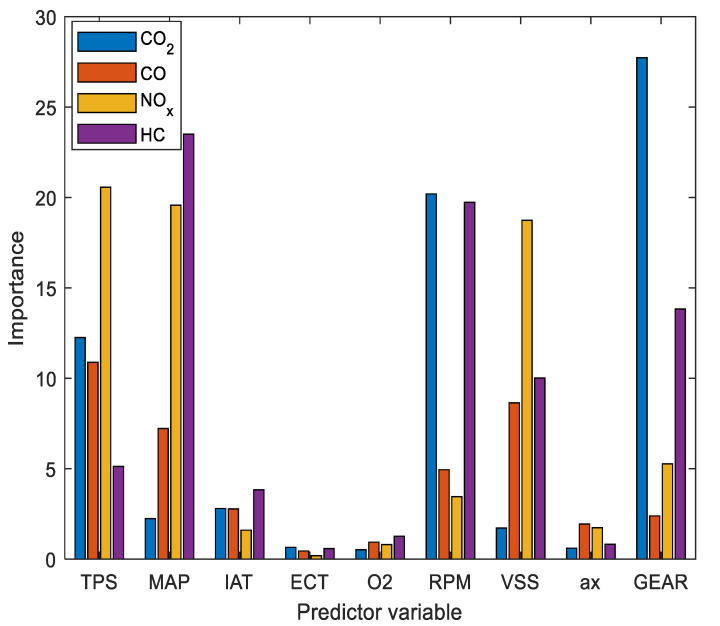
Importance of predictors in instantaneous pollutant emissions.

For the selection of variables with RF, the data obtained in the RDE of Route 1 are taken, being the inputs of all the operating parameters of the vehicle and the outputs the pollutant emissions produced. The result of the most influential predictors is shown in Figure 7.

The most influential variables in pollutant emissions are the TPS, MAP, RPM, VSS and GEAR, leaving aside factors such as IAT, ECT and O_2_, with the level of importance of the cut-off value fixed in 5. Acceleration (*a_x_*) is one of the least influential in a direct way that can be explained by the correlation with VSS and GEAR [16].

### 2.4. Training and Validation of the Neural Network with the Most Significant Variables of Route 1

The data obtained in Route 1 of the RDE test are used to train 1 ANN per pollutant, the ones that have 4 neurons in the input layer, 10 in the hidden layer and 1 in the output layer. Their input vectors, respectively, are:

*CO_2 i_* = [*GEAR_i_, RPM_i_, TPS_i_, MAP_i_*] (11)

*CO_i_* = [*TPS_i_, MAP_i_, VSS_i,_ RPM_i_*] (12)

*NO_x i_* = [*TPS_i_, MAP_i_, VSS_i_, GEAR_i_*] (13)

*HC _i_* = [*MAP_i_, RPM_i_, GEAR_i_,VSS_i_*] (14)

### 2.5. Validation of the ANN with Route 2 Data

**Figure 8:**
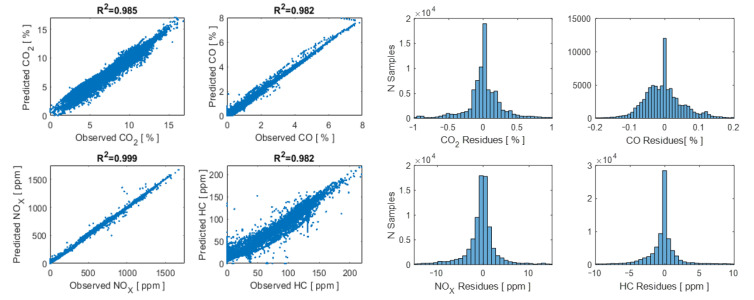
Validation of the ANN with the data from the RDE test Route 2.

The data obtained in Route 2 of the RDE test are applied as inputs to the generated networks, and it can be observed that the adjustment is highly satisfactory, according to the spreading and distribution diagrams of the errors. The residues of the model show a symmetric quasi-normal behaviour around 0, with no offsets in the estimation of each one of the pollutants. The residues behave completely randomly, so inference from other not considered variables in the training of the ANNs is dismissed, as shown in Figure 8.

### 2.6. Double Validation of the ANN. Data Set of 1218.9 km

The 1218.9 km data set was randomly obtained in real driving conditions. The datalogger was kept connected in the vehicle for one month, where three drivers made use of the vehicle without any prior driving instruction to ensure that the data obtained were as realistic as possible. The driving cycles generated were random, without urban, rural or motorway route planning.

### 2.7. Processing and Presentation of Results

From the total travelled distance, 295 files are obtained, one for each driving cycle, which is defined as the travelled distance of the vehicle from the moment the engine is started until the engine speed is below 50 RPM and the vehicle speed is equal to 0 km/h [20]. Likewise, each cycle is subdivided into movement areas and stop areas, considering a driving micro-cycle as the travelled distance executed from one stop area to the beginning of the next one, according to what is shown in [29], where a total of 2785 files are generated under these conditions.

A matrix *Mc_n,m_* is stored per each driving micro-cycle, where *n* represents the number of cycle from which the microcycle *m* was obtained. This matrix contains all the operating and driving parameters shown in Table 1, the selected gear, and the CO_2_, CO, NO_X_ and HC [g/s] instantaneous emission values calculated through the ANNs obtained and validated in Section 2.4 and Section 2.5.

The emission of each pollutant, travelled distance, average speed and time spent on the route travelled are estimated in each micro-cycle matrix per each selected gear.

The environmental conditions do not show great variations throughout the sampling period, since the city of Cuenca is located in the equatorial zone where the climate is practically constant, therefore its influence on the obtained results are discarded.

## 3. Results and Discussion

The data obtained in 1218.9 km of random travel distance through the urban, rural and motorway areas of the city of Cuenca, in a total of 47.06 h, are applied to the models generated, producing a data set of 2,505,459 × 18 data, whose results are shown in Table 4.

**Figure 9:**
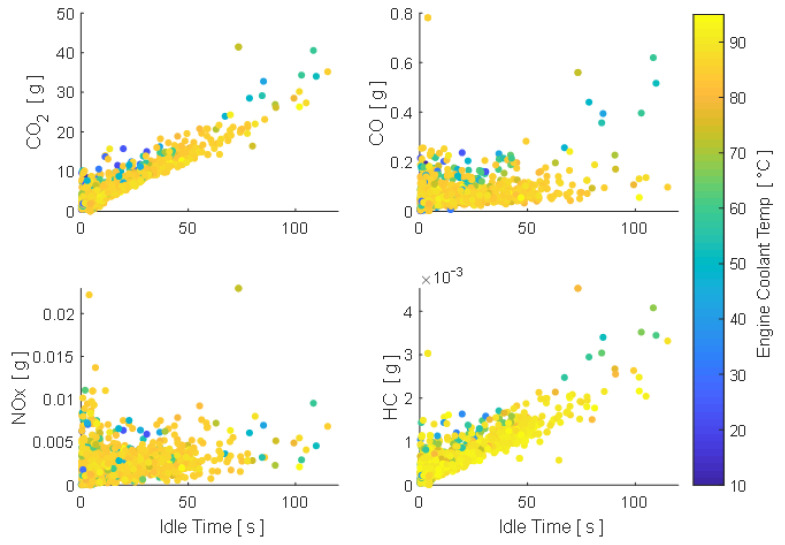
Pollutant emissions generated in idle time.

The results obtained allow evaluating vehicle performance in urban, rural and motorway driving. Stops are considered as periods wherein vehicle speed is less than 1 km/h as specified in [20]. Idle time of the vehicle comprises 14.26% of the total running time, so therefore, emissions generated during stops are: CO_2_ = 9039.2 g, CO = 99.91 g, NO_X_ = 3.54 g and HC = 0.9398 g, at a generation rate of 374.04 mg/s, 4.13 mg/s, 0.146 mg/s and 0.039 mg/s respectively, as shown in Figure 8. The relative idling emissions regarding the total generated during the whole analyzed period correspond to 7.35% of CO_2_, 1.51% of CO, 1.85% of HC and 0.38% of NO_X_. These results do not consider special engine operations during a cold start, which require specific studies in future papers; in this case, the increase of emissions at low temperatures is due to the increase in engine speed and does not consider the enrichment of the mixture, as shown in Figure 9.

During vehicle real driving, the emissions generated depend on the parameters specified in Section 2.3, so therefore, these results are influenced by the different operating conditions of each trip [17] and consider congestion real conditions that [1,30] defined as very important for estimation in models based on average speed.

**Figure 10:**
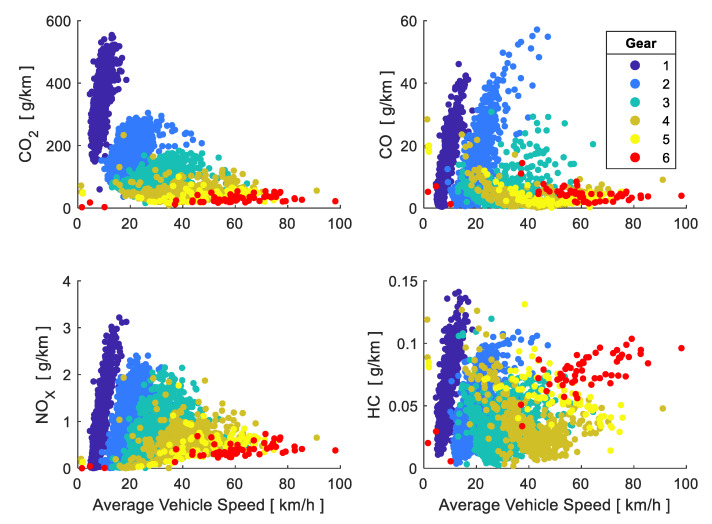
Emission factors according to average speed and gear used.

Figure 10 shows that the 1st, 2nd and 3rd gears are mainly used during the start-up and low average speeds, in short distances travelled mostly in urban areas and very rarely in rural and motorway areas. Emission factors of CO_2_, CO, NO_X_ and HC perform proportionally to vehicle average speed during the period of time where these gears were used, indicating that the lower the speed at which the gear change is made, the lower the pollution generated by the vehicle; for example, the emission factor of CO_2_, CO, NO_X_ and HC at an average speed of 12.96 km/h, may turn, when changing from first to second gear, from [554.17, 46.11, 3.21, 0.141] into [121.98, 7.43, 1.612, 0.076] respectively. From 23.14 km/h on (average speed in urban areas), CO_2_, CO and NO_X_ emissions decrease when the average speed lowers while changing to a higher gear, while HC emissions increase when ascendant changes are made and average speed increases.

Several studies have highlighted the gap existing between emissions produced in real driving, both the ones determined in certification tests [3] and those estimated by different models [31]. The differences in the estimation that are shown when using IVE model are due to factors like vehicle characteristics, wherein parameters such as manufacturing standard, legislation, gas treatment and feed system technologies, trip characteristics, fuel and driving, plus weather conditions stand out [32]. For the estimation of emission factors applying the IVE model, the average speed values for each gear, which are shown in Table 4, are used.

The proposed model determines the emission factor by relating the total amount of pollutant generated and the travelled distance using Equations (9) and (10) according to the average driving speed per gear.

**Figure 11:**
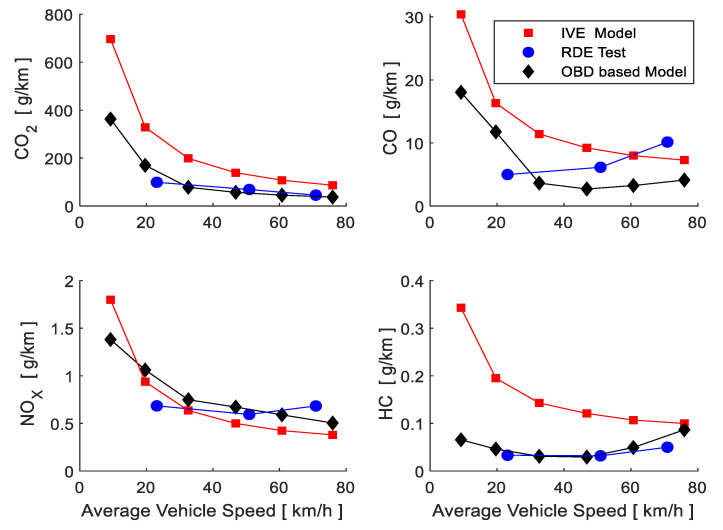
Emission factors according to average driving speed for the IVE model, RDE test and OBDM.

Figure 11 shows the results of the emission factors obtained from the IVE model, RDE test and from the model based on OBD data (OBDM). During urban driving, average driving speed in the RDE test is 23.14 km/h, which is a value extremely influenced by travelled distances made at relatively high speeds in urban areas, so therefore, emissions generated at low driving speeds become less representative, ensuring that emission factors estimated through IVE and OBDM at low driving speeds are higher than the ones determined by the RDE test. Based on this, CO_2_ and NO_X_ emissions, which were determined by the three models, are highly similar. HC emissions determined by RDE and OBDM have highly similar values and behaviors, both lower than what was estimated by IVE. The behavior of CO estimated by RDE and OBDM grows when increasing driving speed, contrary to what is determined by IVE.

The average emission factors for each model, determined from the total emission of the pollutant and total travelled distance, are shown in Table 5, wherein great similarity is present in the RDE and OBDM results. The values estimated by IVE are higher than the other models analysed. The main difference is the CO_2_ emission factor, which, as already analysed, is strongly influenced by low driving speeds in urban areas.

The obtained results from RDE and OBDM are very similar because both are based on measurements in real driving conditions; the RDE model proposes that the data be taken in a proportion of travel that is close to 34%, 33% and 33% compared to 58.27%, 29.26% and 12.29% in urban, rural and motorways, respectively, that fed the OBDM model and is shown in Table 6. One result is that there is a greater amount of data in the urban area, which is where the CO_2_ emission is higher (Figure 11) and that there is less data on the route on the motorway where emissions are lower, causing the average emission value to rise with respect to that obtained by RDE. The idle time values are similar, so they do not contribute to the difference between models.

## 4. Conclusions

This article proposes a method for the estimation of pollutant emissions by applying machine learning to an important set of OBD data. A classifier was initially obtained for the evaluation of the gear selected by the driver based on obtaining labels by K-means with an effectiveness of 99.5% and the subsequent training of a classification tree. The biggest errors occur in the small instants that transition lasts between gears. The calculation of pollutant emissions was made with the most important predictors based on the training of the 4 ANNs from the data of measurement campaigns on two routes executed with measuring devices in the RDE test. The coefficients of determination R^2^ of the 4 ANNs: 0.985, 0.982, 0.999 and 0.982 for the estimation of CO_2_, CO, HC and NO_X_, respectively, which together with the analysis of the residues, allow to highlight the strength of statistical modelling.

Vehicle stops comprise 14.26% of the total driving time, so therefore, emissions generated in this operating condition correspond to 7.35% of CO_2_, 1.51% of CO, 1.85% of HC and 0.38% of NO_X_ regarding total emissions generated during the entire travelled distance of the itinerary. These amounts may vary during in-cold operating, a problem that has not been addressed in this research and need the development of future work.

Average driving speeds in urban driving are low, producing a predominant use of the 1st, 2nd and 3rd gears with the consequent increase in pollutant emission factors. In this point, the proposed model has more strength towards different driving conditions and driving styles in urban area, as it is based on the results of random driving of 712.39 km, compared to the 21.63 km of the RDE test and the results of the IVE model.

When the average driving speed increases, the OBDM and RDE test results are highly similar due to the lower influence of traffic on vehicle performance and the lower amount of temporary driving events.

The obtained model is stronger in different driving conditions and shows better results at low average driving speeds than IVE and RDE models; therefore, it is recommended to be used for the calculation of emission and estimation factors of vehicular emission inventories.

In future developments, the model obtained can be adjusted to different parameters such as vehicle age, driving styles, gradient driving, weather condition and in-cold operating, given that under these operative conditions, the engine control system opts for special operating strategies that directly affect the performance of the emissions generated. The proposed methodology must be replicated in those vehicle models with the greatest presence and activity in the vehicle fleet of the city, with the purpose of being able to adjust the results of vehicular emission inventories.

## Figures and Tables

**Table 1:** Operating and driving parameters.

Parameter	Symbol	Min	Max	Unit
Throttle Position	TPS	0	100	[%]
Mass Air Flow	MAF	0	655.35	[g/s]
Manifold Absolute Pressure	MAP	0	255	[kPa]
Intake Air Temperature	IAT	−40	215	[°C]
Engine Coolant Temperature	ECT	−40	215	[°C]
Vehicle Speed	VSS	0	255	[km/h]
Engine Speed	RPM	0	16,383.75	[RPM]
Acceleration	a_x_	-	-	[m/s^2^]
Fuel flow	ff	0	120	[l/h]
Latitude	Lat	-	-	[°]
Longitude	Long	-	-	[°]
Altitude	Alt	-	-	[m]

**Table 2:** Trip characteristics for the RDE test.

RDE Trip Characteristics	Route 1	Route 2	RDE Trip Requirements	Unit
Sample number	85,697	73,422	-	-
Total distance	62.49	55.41	-	[km]
Total duration	96.99	81.88	90–120	[min]
Urban distance	21.63	17.87	>16	[km]
Rural distance	21.24	18.77	>16	[km]
Motorway distance	19.61	18.76	>16	[km]
Urban distance share	34.61	32.25	29–44	[%]
Rural distance share	34.01	33.87	23–43	[%]
Motorway distance share	31.38	33.88	23–43	[%]
Urban average speed	22.49	23.14	-	[km/h]
Rural average speed	50.14	50.91	-	[km/h]
Motorway average speed	85.19	70.91	-	[km/h]
Urban idle time	11.61	14.45	10–30	[%]
Altitude difference between departure and arrival	−4.4	54	<100	[m]

**Table 3:** RDE test results.

F	Urban [g/km]	Rural [g/km]	Motorway [g/km]	Average [g/km]
CO_2_	83.78	79.20	45.57	70.23
CO	2.85	6.65	6.63	5.33
NO_X_	0.6694	0.8063	0.6820	0.7199
HC	0.0314	0.0426	0.0736	0.0485

**Table 4:** Real driving conditions results.

Gear	Distance [km]	Rate Distance [%]	Time [min]	Rate Time [%]	Average Speed [km/h]
N	2.09	0.17	402.77	14.26	0.31
1	53.61	4.40	348.37	12.34	9.23
2	191.93	15.75	586.25	20.76	19.64
3	464.76	38.13	855.21	30.29	32.61
4	284.71	23.36	364.77	12.92	46.83
5	119.88	9.84	118.41	4.19	60.74
6	101.92	8.36	80.5	2.85	75.97

**Table 5:** Average Emission Factors.

F	IVE [g/km]	RDE [g/km]	OBDM [g/km]
CO_2_	208.97	70.23	111.47
CO	11.83	5.33	6.04
NO_X_	0.661	0.7199	0.777
HC	0.1477	0.0485	0.043

**Table 6:** RDE-OBDM comparison.

Characteristics	RDE				OBDM			
	Idle	Urban	Rural	Motorway	Idle	Urban	Rural	Motorway
Distance [km]	0	21.63	21.24	19.61	2.09	712.39	356.63	149.87
Rate Distance [%]	0	34.61	34.01	31.38	0.17	58.27	29.26	12.29
Time [min]	11.26	57.7	25.41	13.81	402.77	2192.6	435.81	127.86
Rate time [%]	11.61	50.41	22.21	11.15	14.26	79.55	15.81	4.64
Average Speed [km/h]	0	22.49	50.14	85.19	0.31	19.43	49.09	70.32

## Data Availability

Not applicable.
